# Are There Neurophenotypes for Asthma? Functional Brain Imaging of the Interaction between Emotion and Inflammation in Asthma

**DOI:** 10.1371/journal.pone.0040921

**Published:** 2012-08-01

**Authors:** Melissa A. Rosenkranz, William W. Busse, John F. Sheridan, Gina M. Crisafi, Richard J. Davidson

**Affiliations:** 1 Waisman Laboratory for Brain Imaging and Behavior, University of Wisconsin-Madison, Madison, Wisconsin, United States of America; 2 Department of Medicine, University of Wisconsin-Madison, Madison, Wisconsin, United States of America; 3 Division of Oral Biology and Institute for Behavioral Medicine Research, Ohio State University, Columbus, Ohio, United States of America; 4 Laboratory for Affective Neuroscience, University of Wisconsin-Madison, Madison, Wisconsin, United States of America; Leiden University Medical Center, The Netherlands

## Abstract

**Background:**

Asthma is a chronic inflammatory disease noteworthy for its vulnerability to stress and emotion-induced symptom intensification. The fact that psychological stress and mood and anxiety disorders appear to increase expression of asthma symptoms suggests that neural signaling between the brain and lung at least partially modulates the inflammatory response and lung function. However, the precise nature of the neural pathways implicated in modulating asthma symptoms is unknown. Moreover, the extent to which variations in neural signaling predict different phenotypes of disease expression has not been studied.

**Methods and Results:**

We used functional magnetic resonance imaging to measure neural signals in response to asthma-specific emotional cues, following allergen exposure, in asthmatics with a dual response to allergen challenge (significant inflammation), asthmatics with only an immediate response (minimal inflammation), and healthy controls. The anterior insular cortex was differentially activated by asthma-relevant cues, compared to general negative cues, during the development of the late phase of the dual response in asthmatics. Moreover, the degree of this differential activation predicted changes in airway inflammation.

**Conclusions:**

These findings indicate that neurophenotypes for asthma may be identifiable by neural reactivity of brain circuits known to be involved in processing emotional information. Those with greater activation in the anterior insula, in response to asthma-relevant psychological stimuli, exhibit greater inflammatory signals in the lung and increased severity of disease and may reflect a subset of asthmatics most vulnerable to the development of psychopathology. This approach offers an entirely new target for potential therapeutic intervention in asthma.

## Introduction

Airway inflammation in asthma is regulated by complex and diverse mechanisms, with variable disease expression, leading to the emergence of distinct phenotypes. As currently envisioned, each phenotype is characterized by an underlying physiological pathology, indicating a need for different treatment approaches [1], [2]. In addition to the pathological cellular and molecular immune processes that define the currently described phenotypes, evidence also suggests that neural processes, particularly those involved in emotion, contribute to the regulation of airway inflammation and clinical expression of disease severity in asthma [3], [4], [5], [6], [7], [8], [9]. Here, we present evidence to indicate that the current characterization of asthma phenotypes by peripheral immune processes, should be expanded to include functional differences in affective neural circuitry, which we define as a neurophenotype.

The word *phenotype* refers to the observable properties of an organism that result from the interaction between genes and the environment. In this instance, a *neurophenotype* refers to the pattern of symptom expression that results from an interaction between immune responses that regulate inflammation and the neural “context”. The need to expand the purview of factors that contribute to disease expression in asthma to include the neural context is reflected by observations that asthma symptoms and severity are increased during periods of increased stress or emotion. For example, we have previously demonstrated that undergraduate asthmatic subjects had greater airway inflammation and airflow obstruction in response to allergen challenge during final examination week compared to an identical challenge during a relatively stress-free period [3].

Although this is not a new concept, the possibility of neural regulation of inflammation in asthma is a novel concept. A broad array of evidence establishes bi-directional, causal links between inflammation and psychological state [4], [10] that, in asthma, may be reflected in the two-fold increase in risk for development of anxiety and depressive disorders [11]. This co-morbidity comes at a high cost, as depression and anxiety may lead to decreased asthma control, greater frequency of symptoms and increased healthcare utilization [12]. These observations clearly implicate neural involvement in the modulation of airway events in asthma, since it is the brain that must transduce stress- and emotion-related stimuli from the environment, and then in turn signal inflammatory mechanisms in the lung to further exacerbate underlying disease. Indeed, others have demonstrated impairment in the neural processing of respiratory events using EEG, in individuals with asthma [13], [14]. Nonetheless, research directed toward understanding the pathophysiology of asthma and the expression of asthma symptoms has been focused almost exclusively on the lung.

In our previous work, the anterior cingulate cortex (ACC) and insula were identified as two likely constituents of a circuit that plays a key role in the regulation of inflammation and modulation of lung function [8] in asthmatic individuals with a dual-phase response to inhaled allergen. Anatomical connectivity of the ACC and insula suggest that they are involved in bidirectional communication with peripheral autonomic and immune targets and thus were the focus in this study [15], [16]. The current investigation builds on our previous work by extending our research over a continuum of disease severity to determine if neural activation can predict the emergence of inflammation and airway obstruction following an acute exposure to allergen. To accomplish this goal, we studied individuals with isolated immediate responses to allergen, presumably reflecting less severe asthma, and dual-responders who have greater inflammatory responses, i.e., more severe asthma, as well as healthy controls. In this way, we were able to determine if the neural context following allergen exposure predicts who will go on to have a dual response.

## Methods

### Experimental Design

The inhalation of an allergen causes pulmonary mast cell activation, degranulation and mediator release to rapidly contract bronchial smooth muscle during the early phase of an allergic asthmatic reaction in the airway. This phase typically resolves and lung function returns to normal within an hour. However, a subset of individuals have a dual response – the early-phase response followed by a late-phase reappearance of airway obstruction 4–8 h later – which is related to the development of airway inflammation, reflected by recruitment of eosinophil (EOS) and basophil progenitors from bone marrow [17]. Unlike the bronchospastic nature of the early phase, late-phase airway obstruction is attributed largely to the development of inflammation.

The current study was designed to determine if activity in the ACC and insula is associated with systematic alterations in lung function and inflammatory mediators. Using functional magnetic resonance imaging (fMRI), we compared the neural response to asthma-relevant emotional stimuli of individuals who experience a late-phase response to allergen (LPR) to that of those who experience only an early response (NLPR) to determine if the sensitivity of these brain regions to disease-relevant information during the development of inflammation can distinguish individuals with a dual response to inhaled allergen. We predicted that emotional neural circuitry would show a greater response in LPRs than in NLPRs or healthy controls, to asthma-related information (As) compared to negative (Ng) or valence-neutral (Ne) stimuli due to the greater inflammatory response. Further, this greater responsivity should be specific to allergen challenge (Ag), during which greater inflammation is present. In addition, we predicted that individual differences in this differential neural activation would be associated with the magnitude of the peripheral physiological responses to Ag. If so, these circuits may not only be responsive to physiological changes associated with allergen exposure, they may contribute to the perpetuation of the inflammatory response that is observed hours and even days after exposure to allergen in the late-phase response [18].

**Figure 1:**
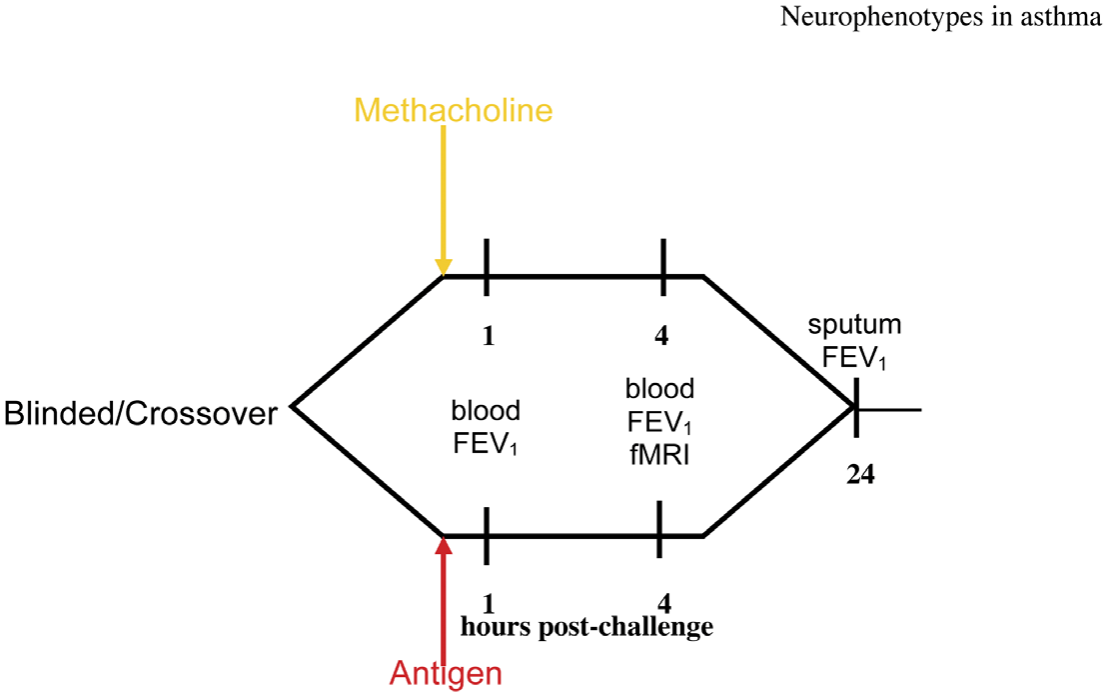
Experimental Design. Timing of experimental challenges and measures collected. Participants underwent both Ag and Meth challenges separated by at least 4 wk, in a complete within-subjects crossover design. Challenge order was counterbalanced and double-blind.

Asthmatic and non-asthmatic control participants each underwent two inhalation challenges (figure 1), separated by a minimum of 4 weeks: Methacholine (Meth), a cholinergic agonist, causes acute bronchoconstriction without significant airway inflammation; and subject-specific Ag that causes an immediate or dual response in susceptible individuals. As in our previous study [8], Meth was used as a control challenge to evaluate the contribution of airway constriction and chest tightening, without inflammation, as contributors to the hypothesized effects of Ag. Order of administration of Meth and Ag was counterbalanced and double-blind. For each challenge, fMRI scans were performed at 4 h post-challenge, timed to coincide with the onset of the late-phase response to Ag. During neuroimaging, participants performed a variation of the Stroop task [19] where they identified the color of As, Ng or Ne words. Briefly, the Stroop task is an index of cognitive interference where reaction time to identify the color of a word with a salient meaning is delayed to the extent that the meaning of the word draws cognitive resources. Lung function and local inflammatory potential were measured before, during and after challenge.

### Participants

Eighteen participants (11 female, *M* age = 22.53) with mild allergic asthma and ten non-asthmatic, non-allergic controls (6 female, *M* age = 23.2) were recruited for study. All asthmatic participants demonstrated an early-phase response (>20% decrease in FEV_1_ within 1 h of Ag challenge) during screening. Those showing an LPR exhibited a second decrease in FEV_1_ (>15%) 4–8 h post-Ag challenge. Of the eighteen asthmatic participants, ten experience an LPR. Controls showed no decrease in FEV_1_ in response to challenge. During the study, no participants required inhaled corticosteroids. Participants were instructed to withhold their medication for every study visit. Written informed consent was obtained from all study participants. This study was approved by the Institutional Review Board of the University of Wisconsin-Madison.

### Lung Function and Inflammation Measurement

Lung function (FEV_1_) was measured according to American Thoracic Society standards [20] using portable spirometry equipment. Response to challenge was computed as the percent peak fall in FEV_1_, from saline baseline levels, following administration of Meth and Ag. For the early phase, the peak fall was the lowest FEV_1_ value obtained immediately following challenge and for the late phase, the peak fall was the lowest FEV_1_ measured anytime during the 8 h following recovery from the early response.

Inflammatory markers were determined by measuring the total white blood cell count and differentials in induced sputum samples. Sputum inductions were performed as previously described [21], before and 24 h post-challenge and were reported as the percentage of monocytes/macrophages, lymphocytes, neutrophils and EOS. Sputum was not induced immediately following the LPR because albuterol is given prior to induction and would interfere with the full expression of the LPR.

### Inhalation Challenges

Allergen challenges were performed using an allergen to which the subject has a positive skin test and were limited to extracts of house dust mite, ragweed, and cat dander. Briefly, subjects inhaled nebulized escalating doses of allergen extract (Ag) or Provocholine™ (Meth). Spirometry was performed after each dose and was stopped when FEV_1_ decreased by at least 20% compared to baseline. Control subjects received the 6 most concentrated of 10 ragweed extract doses.

### Brain Image Acquisition

A GE Signa 3.0-Tesla high-speed imaging device with a quadrature head coil (General Electric Medical Systems, Milwaukee, Wisconsin) was used to acquire both anatomical and functional images. Two runs of functional images consisted of 30-×4-mm sagittal EPI slices covering the whole brain (1-mm interslice gap; 64×64 in-plane resolution, 240-mm FOV; TR/TE/Flip = 2000 ms/30 ms/60°). Immediately preceding acquisition of functional images, a high-resolution T1-weighted anatomical scan (3D T1-weighted inversion recovery fast gradient echo; 256×256 in-plane resolution, 240 mm FOV; 124×1·1 mm axial slices) was acquired.

### Imaging Task

The stimuli were 20 As (e.g., wheeze), 20 Ng (e.g. loneliness) and 20 Ne (e.g. curtains) words presented digitally, in one of four colors. Asthma-related stimuli were words associated with an asthmatic episode, generated by individuals with asthma. Ng and Ne words were selected from the ANEW dataset [22]. Each set was matched on word length, usage frequency and part of speech. Stimuli were presented using the Avotec Goggle System (Avotec, Inc., Stuart, FL). We instructed participants to identify the color of each stimulus by pressing one of four buttons on an MRI-compatible response pad (Current Designs, Inc., Philadelphia, Pennsylvania). The task and the color-button associations were learned prior to the first scanning session and were practiced immediately before the first functional scan. Reaction time (RT) and accuracy were recorded using E-Prime software. In each run, 30 stimuli (10 per category in random order) were presented for 2 s each, with a pseudo-randomized inter-stimulus interval of 8–12 s.

### Data Preprocessing

All data processing was done using AFNI software [23]. Following image reconstruction, each time series was corrected for motion by realigning it with the first stably acquired image. Individual subject data for each run were then analyzed using a general linear model (GLM) with separate regressors for each experimental condition (i.e., As, Ng and Ne), formed by convolving a stimulus boxcar function with an ideal hemodynamic response function. Six dimensions (inferior to superior, anterior to posterior, left to right, yaw, pitch and roll) of motion were also modeled as covariates of no-interest. The GLM yielded a set of contrast maps (As-Ne, Ng-Ne, As-Ng, As-fixation, Ng-fixation, Ne-fixation) for each individual. Differences between subjects in overall scaling of fMRI data were removed by converting the parameter estimates of the contrast maps to percent signal change by expressing the estimated signal change for a given contrast as a percentage of the baseline signal. These contrast maps were then transformed into standard (MNI) space and spatially blurred using a 5 mm full-width-at-half-maximum Gaussian spatial filter.

### Statistical Analyses

Repeated measures analysis of variance (ANOVA) was used to evaluate the effects of group and challenge on peripheral inflammatory markers and behavioral performance on the Stroop task. Significant interactions and main effects were followed-up with t-tests for pair-wise comparisons.

A mixed-model ANOVA was used to examine the effects of group, challenge, and word valence, their interactions and select simple effects on neural activity. A whole-brain search was conducted to identify regions with activation showing a main effect of group, a challenge x group interaction, or a challenge x group x valence interaction. Monte Carlo simulation was used to correct for multiple comparisons, based on volumetric criteria (see Supplementary Information S2 for details).

Individual differences analyses examining relations between neural activation and peripheral measures were also conducted. We predicted that individual differences in the magnitude of insula response to As stimuli during Ag challenge would be associated with changes in the peripheral measures of lung function and inflammation. To this end, we tested the correlation between neural activation and peripheral measures, across groups. For each participant, difference images were created for each valence by subtracting the contrast map for the Meth challenge from the corresponding contrast map for the Ag challenge (e.g., [As-Ng] for Ag - [As-Ng] for Meth). We conducted a whole-brain voxel-wise search for regions showing associations with peak fall in FEV_1_ (Ag-Meth) and percent EOS (Ag-Meth). For a description of missing data, see Supplementary Information S1. Correction for multiple comparisons was performed as described in Supplementary Information S2.

## Results

### Peripheral Measures

#### Pulmonary function responses

**Figure 2:**
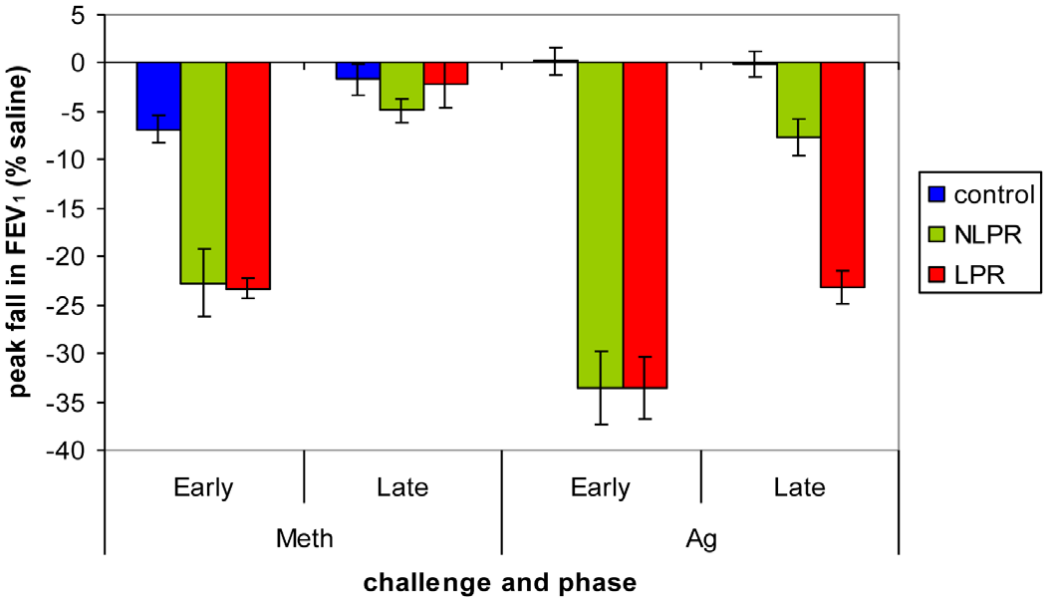
Peak fall in FEV_1_ following Meth and Ag challenge. Group x time x challenge interaction for peak fall in FEV_1_ (F(2,24) = 8.92, *p* = .001). Following Meth challenge, the LPR and NLPR groups showed a significantly greater early phase peak fall in FEV_1_ than the control group (LPR vs. Control: *t*(17) = −9.28, *p*<.001; NLPR vs. Control: *t*(15) = −4.43, *p*<.001), but the two groups of asthmatics did not differ (*t*(16) = −.17, *p* = .87). Lung function did not differ across groups during the late phase period after Meth challenge (F(2, 24) = .79, *p* = .47). Following Ag challenge, the LPR and NLPR groups again showed similar early phase peak falls in FEV_1_ (*t*(16) = −.01, *p* = .99) that were significantly greater than that of the control group (LPR vs. Control: *t*(17) = −9.40, *p*<.001; NLPR vs. Control: *t*(15) = −8.70, *p*<.001). During the late phase period, however, the LPR group showed a greater peak fall in FEV_1_ compared to both the NLPR (*t*(16) = −6.13, *p*<.001) and control groups (*t*(17) = −10.55, *p*<.001), whereas the NLPR group showed a peak fall between that of the LPR and control group (NLPR vs. Control: *t*(15) = −3.35, *p* = .004). Error bars represent standard error of the mean.

There were no differences among the groups in lung function at baseline (F(2, 25) = 1.92, *p* = .17; LPR: *M* = 92%, *SD* = 9.7% predicted FEV_1_; NLPR: *M* = 95%, *SD* = 5.0%; Control: *M* = 99%, *SD* = 6.7%). A significant group x time x challenge interaction for peak fall in FEV_1_ (F(2,24) = 8.92, *p* = .001) was present, such that following the Meth challenge, LPR and NLPR groups’ lung function was indistinguishable and significantly lower than the control group, during the early-phase response, but the 3 groups did not differ during the late phase. Similarly, both asthma groups showed an identical fall in lung function during the early response to Ag, that was significantly greater than the control group. However, during the late-phase response to Ag, all 3 groups differed with respect to lung function where the LPRs had a large peak fall, the NLPR group had a minor peak fall and the control group had no fall (see figure 2).

#### Sputum analyses

**Figure 3:**
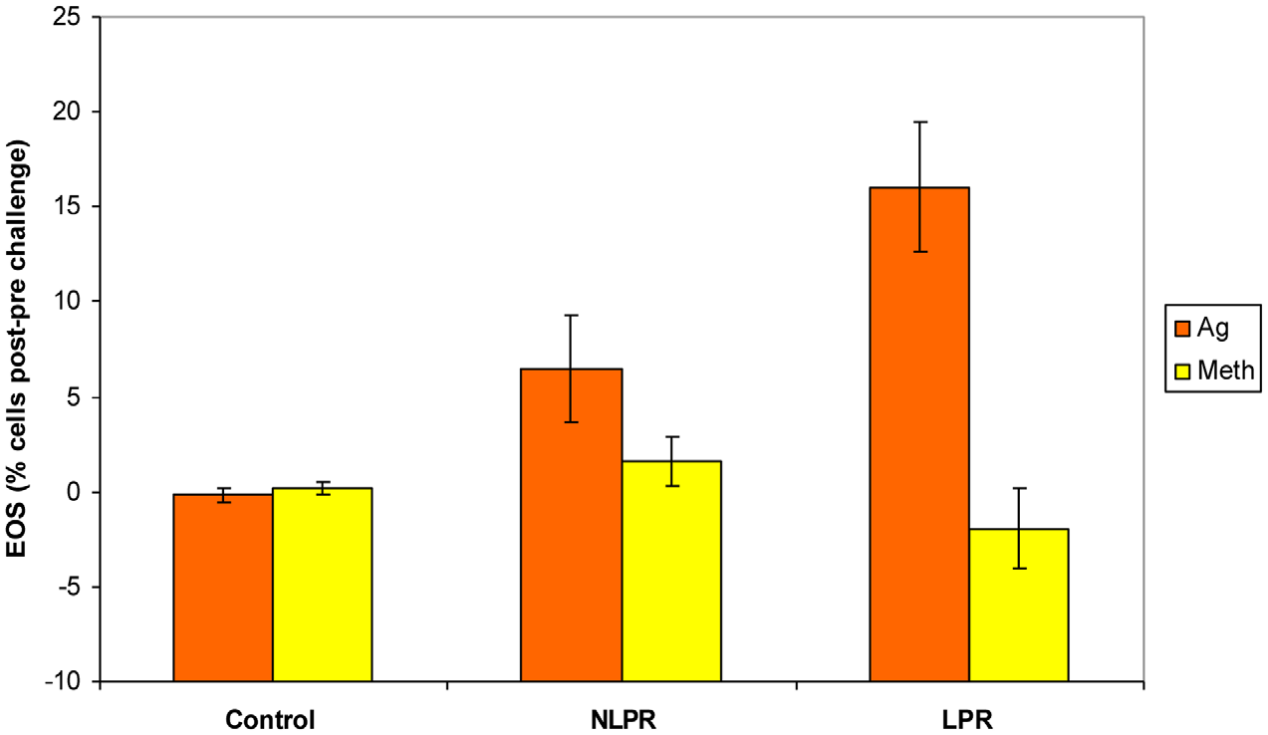
Greater percentage of sputum EOS during Ag challenge in late-phase responders. Percentage of sputum eosinophils 24 h post-challenge relative to pre-challenge (group x challenge interaction; *F*(2,24) = 8.16, *p* = .002). The increase in EOS for the LPR group was greater than that of both the NLPR (*t*(15) = 2·13, *p* = .05) and controls (*t*(17) = 4.95, *p*<.001) following the Ag challenge. The NLPR group showed a small, but significantly greater increase in EOS than controls (NLPR vs. Control: *t*(16) = 2.64, *p* = .018). No group differences were present following the Meth challenge (LPR vs. NLPR: *t*(15) = −1.38, *p* = .19; LPR vs. Control: *t*(17) = −1.07, *p* = .30; NLPR vs. Control: *t*(16) = 1.16, *p* = .26). Error bars represent standard error of the mean.

Sputum EOS, measured 24 h post-challenge, increased significantly only after Ag challenge and only in the two asthma groups (F(2,24) = 8.16, *p* = .002). Further, the increased EOS following the Ag challenge was significantly greater for the LPR group than the NLPR, whereas there was no difference between the two asthma groups following the Meth condition. Both asthma groups differed from the control group following Ag, but not Meth challenge (see figure 3).

### Functional Neuroimaging Measures: ANOVA

**Figure 4:**
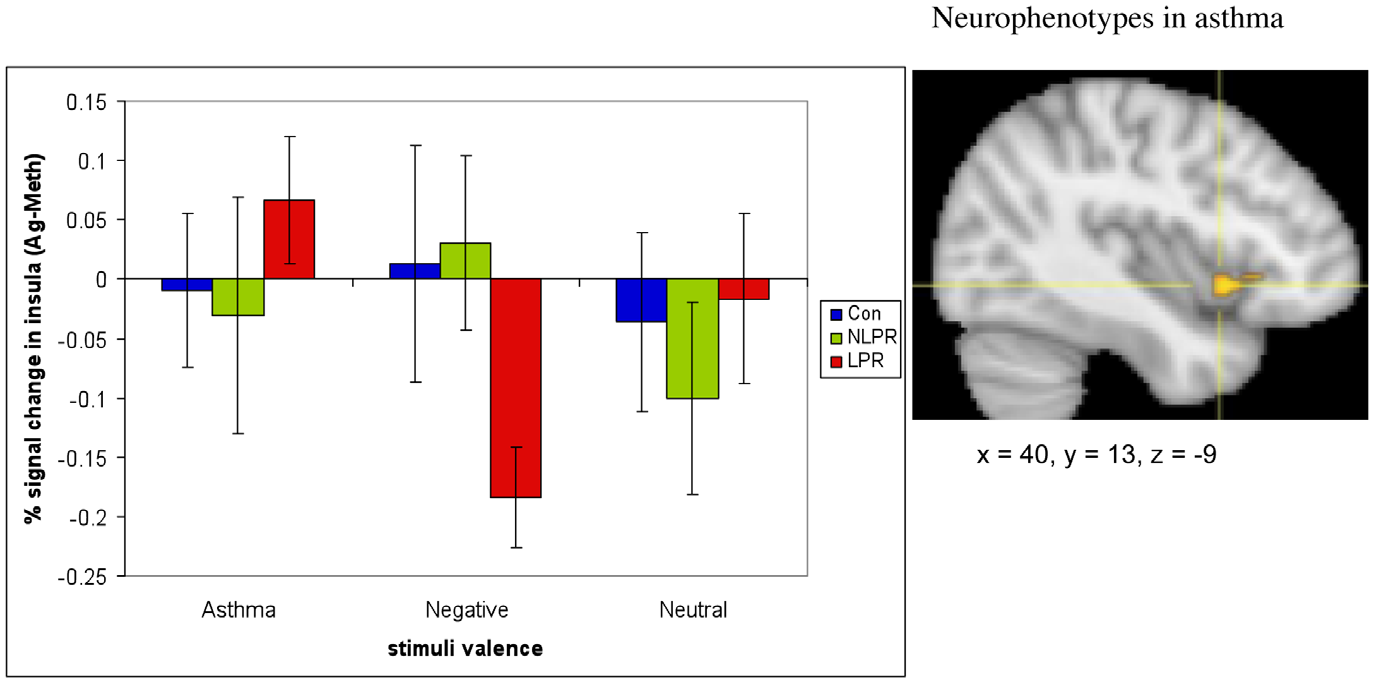
Individuals with asthma who have a late phase response show increased anterior insular reactivity to asthma-related words, during an antigen challenge relative to a methacholine challenge, compared to their response to negative or neutral words. The difference in right anterior insula response to asthma-related versus negative words, during antigen versus methacholine challenge (Ag(As-Ng)-Meth(As-Ng)) in the late phase group was significantly different from the insula response of both the non-late phase group (F(1,15) = 6.47, *p* = .02) and the control group (F(1,17) = 10.53, *p* = .005). (To see % signal change in the insula during individual conditions, see Figure S1). The overall group x challenge x valence interaction was significant (F(4, 48) = 3.24, *p*<.05). Error bars represent standard error of the mean. Coordinates are in Montreal Neurological Institute Space (MNI).

**Figure 5:**
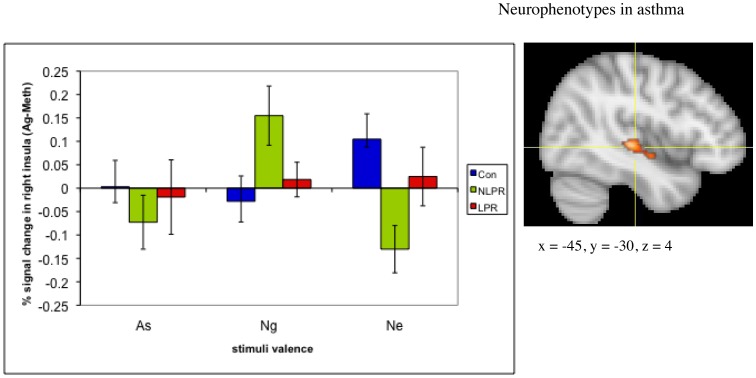
Asthmatic individuals with no late phase response (NLPR) to antigen show increased ventral posterior insular activity to negative words, during an antigen challenge. Ventral posterior insula response to negative words, relative to asthma-related and neutral words, during Ag compared to Meth challenge was significantly greater in the NLPR group than the LPR (As: F(1,15) = 3.31, *p* = .089; Ne: F(1,15) = 12.33, *p* = .003) and control group (As: F(1,16) = 8.82, *p* = .009; Ne: F(1,16) = 12.21, *p* = .003). Error bars represent standard error of the mean. Coordinates are in Montreal Neurological Institute Space (MNI).

The fMRI data revealed a group x challenge x valence interaction in a region of the right anterior insula (616 mm^3^; F(4, 48) = 3.24, *p* = .12 corrected; see figure 4). This cluster did not meet the size threshold necessary to achieve a corrected p-value ≤05. However, we include it because of the specificity of our a priori prediction of this effect in this precise location. Examination of the simple interaction contrasts showed that this effect is driven primarily by a potentiation of the insular response to As words and an attenuation to Ng words during Ag, relative to Meth challenge, in LPRs compared to NLPRs and controls (see Figure S1). In addition, a region of the left ventral posterior insula also showed a group x challenge x valence interaction (1496 mm^3^; F(4, 48) = 4.78, *p*<.01 corrected; see figure 5). However, examination of the simple interaction contrasts reveals that this interaction is primarily driven by a potentiation in responsivity to Ng words and an attenuation to As and Ne words during Ag challenge, relative to Meth challenge, in NLPRs compared to the other two groups, which was not predicted. An exhaustive list of all clusters resulting from the ANOVA analysis, that survived multiple comparison correction or were in predicted regions, can be found in Table S1.

### Functional Neuroimaging Measures: Voxel-wise Regressions with Peripheral Measures

**Figure 6:**
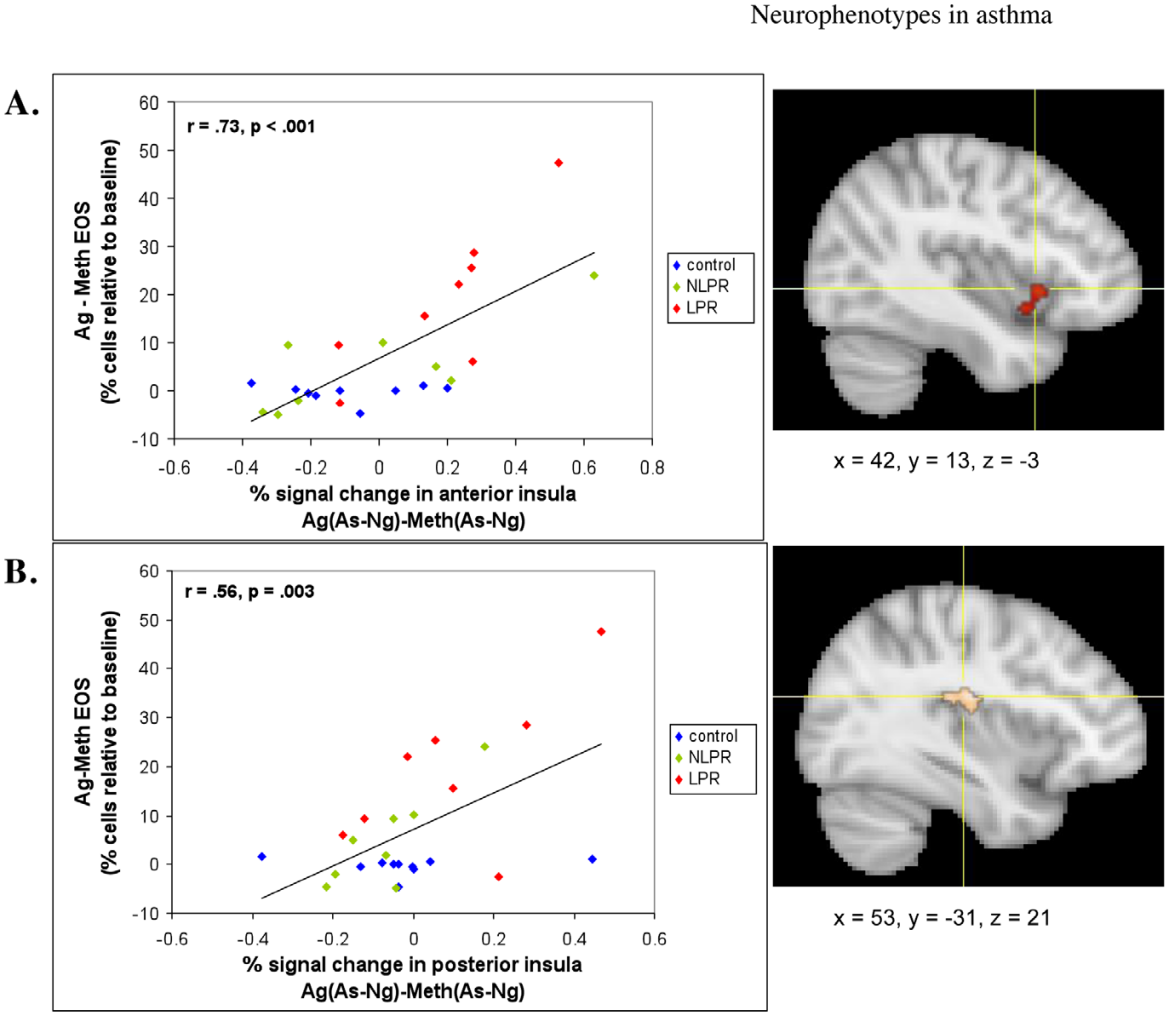
Insula activity predicts peripheral measures of inflammatory potential. Percent signal change in the (a) anterior (*r* = .73, *p*<.001) and (b) posterior (*r* = .56, *p* = .003) insula in response to asthma, compared to negative words, during antigen, relative to methacholine challenge (Ag[As-Ng]-Meth[As-Ng]) and percentage of EOS in sputum during late phase antigen relative to methacholine challenge [Ag-Meth]. Coordinates are in MNI space.

**Figure 7:**
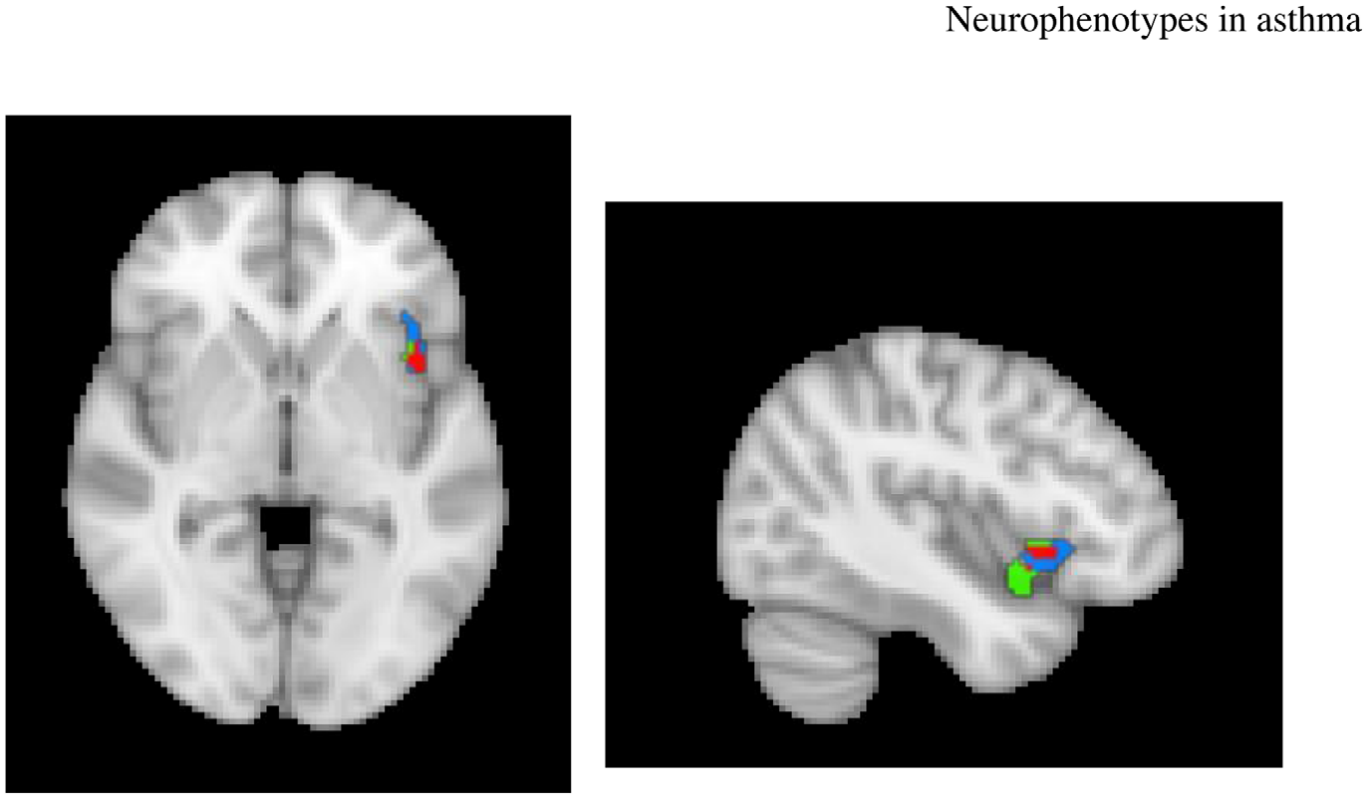
Overlap in voxels in the right anterior insula between the cluster showing a group x challenge x valence interaction and the cluster showing a correlation with increase in sputum eosinophils during Ag challenge. Red voxels indicate the area of overlap; blue voxels belong to only the cluster showing a 3-way interaction; green voxels belong to only the cluster showing the correlation with eosinophils.

The individual differences analyses revealed that the increase in percentage of sputum EOS between the Ag versus Meth challenges was strongly predicted by the corresponding Ag versus Meth difference for the As-Ng contrast in the right anterior insula (1896 mm^3;^ *r* = .73, *p*<.001, corrected) and the right posterior insula (432 mm^3^; *r* = .56, *p*<.01, corrected). When examining these relationships within only those with asthma, the associations are even stronger (*r* = .77, *p*<.001 and.76 *p* = .001, respectively; see Figure S2). The direction of these correlations indicates that subjects with greater percent signal change in the insula, in response to As versus Ng words, show a larger increase in EOS following Ag, relative to Meth challenge (figure 6a & 6b). The anterior insula cluster has significant overlap (208 mm^3^) with the region of the insula that showed a group x challenge x valence effect (see figure 7).

**Figure 8:**
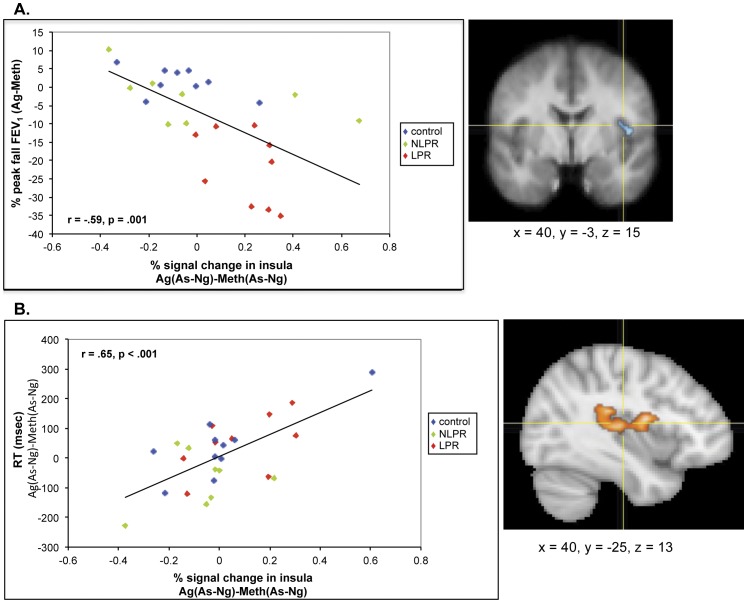
Insula activity predicts magnitude of airway obstruction and cognitive interference of disease-related information during the late phase response to Ag. Percent signal change in the right mid- and posterior insula in response to asthma compared to negative words [As-Ng] and (a) percent peak fall in FEV_1_ [Ag-Meth] (*r* = −.59, *p* = .001) and (b) reaction time to identify the color of As words, relative to Ng words [Ag(As-Ng)-Meth(As-Ng)] (*r* = .66, *p*<.001) during late phase antigen relative to methacholine challenge. Coordinates are in MNI space.

We observed an analogous relationship (*r* = −.59, *p*<.001, corrected) between MR signal in an area of the right mid-insula (Ag(As-Ng)-Meth(As-Ng)) with the change in peak fall from baseline in FEV_1_ between Ag and Meth challenges (488 mm^3^; figure 8a). In the subset of asthmatic participants only, this association remained strong, though less so than in the full sample (*r* = −.53, *p* = .03; see Figure S3a), which isn’t entirely surprising since there is some natural variation in lung function in non-asthmatic individuals. The direction of this relationship indicates that greater percent signal change in the insula in response to As, relative to Ng words, is associated with a greater decrease in lung function, during the Ag compared to Meth challenge. This is the same region of the insula that showed a correlation with both the decline in FEV_1_ and increases in sputum EOS observed in a previous investigation [8].

### Behavioral Performance

**Figure 9:**
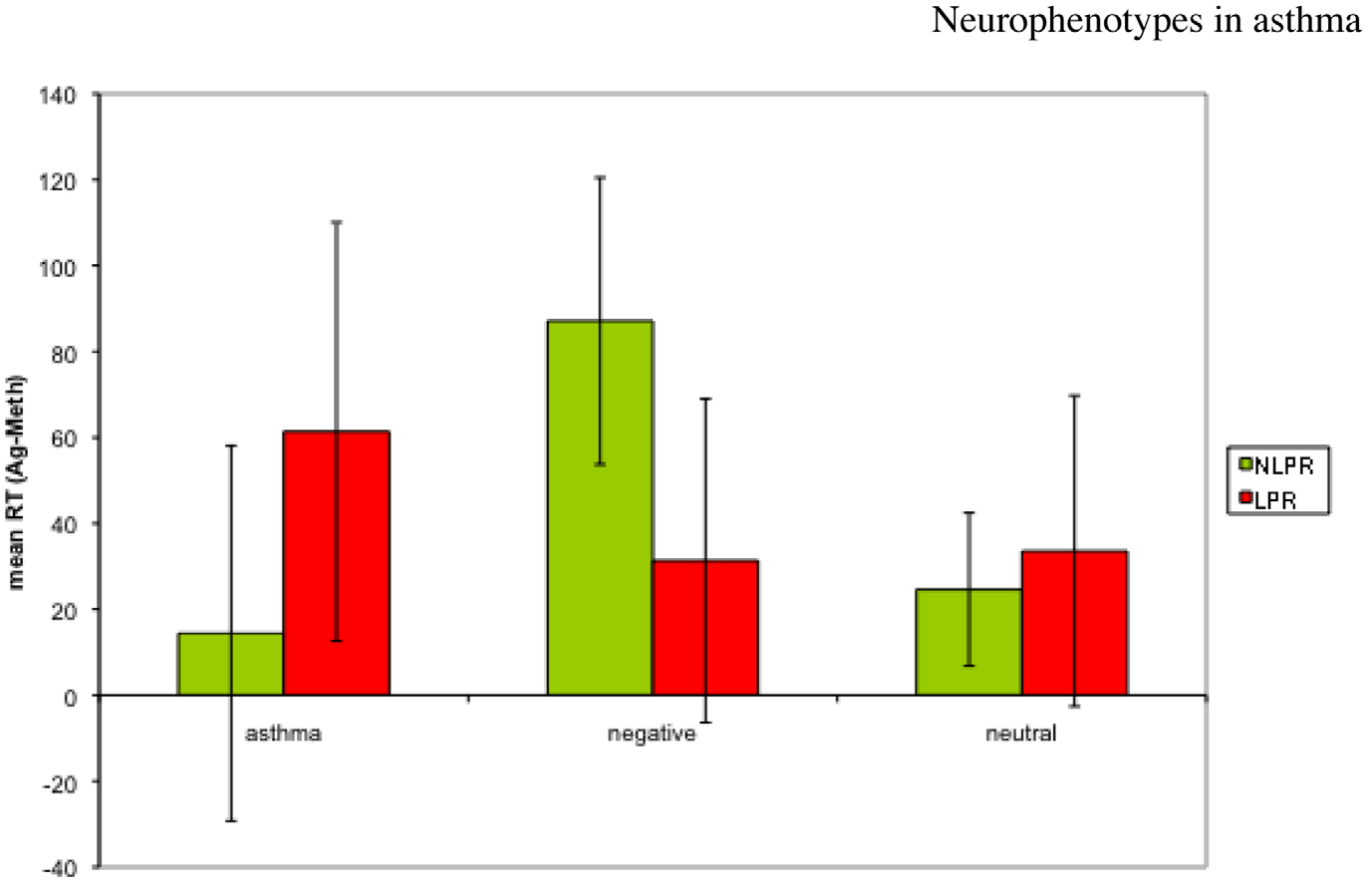
Group x time x valence interaction in reaction time to identify the color of the word stimulus (F(2,14) = 4.46, *p* = .032) among individuals with asthma. The only pair-wise comparison to reach a significance level of *p*<.05 is the difference between the response of the NLPR group to Ng words between Ag and Meth challenge (t(7) = −2.61, *p* = .035).

We found a main effect of valence (F(2.22) = 10.15, *p* = .001) on RT in the Stroop task, where participants (across groups) were faster to respond to asthma words than to either negative (*t*(23) = −2.65, *p* = .014) or neutral (*t*(23) = −4.61, *p*<.001) words. Because the 3-way interaction in insula activity was primarily driven by the difference in neural responsivity between the two asthma groups, we repeated the reaction time ANOVA including only asthmatics. Here we found a group x challenge x valence interaction (F(2,14) = 4.46, *p* = .032), where participants in the LPR group were slower to respond to asthma words during Ag compared to Meth challenge, whereas individuals in the NLPR group were slower to respond to Ng words during Ag compared to Meth (figure 9). The only significant pair-wise comparison in this complex interaction was the difference in response to Ng words in NLPRs between challenges (*t*(7) = −2.61, *p* = .035). Nonetheless, the ability to detect a significant pattern that behaviorally distinguishes these two groups of asthmatics, based on valence and challenge is remarkable given the sample size. A typical sample size for a study finding a similar behavioral effect is more than double that used here [24]. Moreover, this behavioral pattern is consistent with the pattern of neural responses observed in our fMRI data.

### Functional Neuroimaging Measures: Voxel-wise Regressions with Behavioral Measures

Individual difference analyses between fMRI data and RT revealed a positive association (*r* = .65, *p*<.001, corrected) between neural activity (Ag(As-Ng)-Meth(As-Ng) and RT to identify the color of As words, relative to Ng words (Ag-Meth; 4344 mm^3^) in a similar region of the insula as that showing an association with decline in FEV_1 (_ figure 8b). This association in those with asthma alone was somewhat attenuated, but still quite strong (*r* = .55, *p*<.05; see Figure S3b). This suggests that those individuals who show a larger increase in insula activity in response to As vs. Ng words, during Ag challenge, are slower during Ag vs. Meth challenge to identify the color of As words. In other words, this challenge- and content-specific increase in insula activity is associated with greater cognitive interference, as indexed by a relatively delayed behavioral response [25]. A complete list of results from the voxel-wise regression analyses can be found in Table S2.

## Discussion

In the current study, we demonstrated that the anterior insula was differentially sensitive to disease-relevant information, during the development of inflammation and that activity in all 3 subdivisions of this region predicted disease severity as indexed by the magnitude of expression of disease markers. To isolate responses specifically initiated by the onset of an inflammatory airway response from those caused by the development of airway obstruction we contrasted neural and peripheral physiological changes in response to an Ag challenge to those of a Meth challenge. The contrast of the neural response to disease-specific emotional information with that of generally negative and neutral information, during each of these challenge conditions, allowed us to identify brain circuits that were differentially sensitive to asthma-relevant affective stimuli under different physiological challenge conditions. In our sample, LPRs and NLPRs had indistinguishable early-phase airway responses to Ag challenge that diverged during the late-phase period. By comparing these two groups, we were able to determine that neural responses measured in between the early- and late-phase periods – the neural context – predicted the subsequent development of late-phase inflammation and lung function decline, and thus may reflect a distinct neurophenotype, characterized by increased airway inflammation and disease severity as represented by the LPR.

We do not mean to imply that the neurophenotype that we describe here is trait-like. It is likely that, given different environmental circumstances, such as psychological challenges, the pattern of neural activity during Ag challenge, would be different. It is important to not that the task participants performed in the fMRI scanner was not considered a psychological stressor. Rather, it served as a probe of affective neural circuitry under different challenge conditions. Our data demonstrate that the cognitive backdrop on which a physiological stimulus occurs is important in determining the ultimate impact of that stimulus. Thus, an individual with asthma who does not typically have a late-phase response, could progress to an LPR during a period of increased environmental challenge. This was indeed observed during an experimental rhinovirus infection, where 8 of 10 individuals had a late-phase response following infection compared to 1 of 10 individuals before infection [26]. Moreover, our previous work showing a more severe inflammatory response to allergen challenge during final examination period supports this hypothesis [3].

In our current data, activity in the insular cortex best characterized the observed pattern of symptom expression and distinguished those who would go on to have a LPR from those who would not. This region displayed differential activity to disease-relevant emotional stimuli, only during the development of the late-phase response, which was significantly greater in LPRs than in NLPRs. Moreover, the magnitude of this difference in insula responsivity predicted the percentage of EOS in lung sputum 24 h after challenge, as well as the decline in lung function during the late-phase response.

Since fMRI data collection occurred 4 h post-challenge, before the onset of the late-phase decline in lung function, the group difference in insula responsivity could be a consequence of early-phase physiological signals (i.e. afferent signaling). Alternatively, it may reflect neural modulation of the response of the lungs to inflammatory mediators, which determines the subsequent development of the late-phase response (i.e. efferent signaling). We cannot definitively parse the contribution of each of these pathways to the observed pattern of neural response. However, the heightened insula response to As words was specific to blinded administration of Ag. This suggests that the afferent signals preceding or associated with incipient inflammation are necessary. On the other hand, the temporal precedence of fMRI data collection relative to the onset of the late-phase response (both lung function decline and inflammation), suggests that efferent modulation may also be involved. Combined with neuroanatomical evidence discussed below, these observations lead us to speculate that, in all likelihood, the changes in neural activity that we observed reflect both afferent and efferent processes.

Based on its anatomical connectivity, others have suggested that the insula plays a role in the integration of information emerging from different sensory modalities, including the viscera, with cognitive and emotional information to motivate behavioral and physiological homeostatic responses [15], [27], [28], [29], [30], [31]. In primates, the insula receives sensory input from all tissues in the body [27], [32], [33] and can be divided up into roughly three sub-regions: posterior, mid, and anterior. The differences in connectivity of insular sub-regions are suggestive of possible roles in influencing the asthmatic response to inhaled allergen (for a detailed discussion of insular connectivity, see [16]). Because of its high-fidelity sensory input from the thalamus and interconnectivity with the somatosensory cortex [28], [29], a plausible role for the dorsal posterior insula would be to integrate viscerosensory signals associated with exposure to allergen across physiological systems into a cohesive representation (see Supplementary Information S3 for a discussion of the distinction between dorsal and ventral posterior insula). Indeed, we found a large cluster in the posterior insula that showed greater activity in asthmatics (collapsed across groups) than controls, across valence, during the Ag challenge (see Figure S4), suggesting a representation of the homeostatic disturbance associated with the early-phase response.

In the mid-insular region, activity in response to asthma-relevant stimuli following Ag challenge predicted the magnitude of the peak fall in lung function during the late-phase period, a finding that replicates our previous work [8]. In addition, this differential mid-insula activity predicted the relative increase in RT in an analogous contrast (Ag(As-Ng)-Meth(As-Ng). Based on its connectivity with motor cortex and efferent projections to sub-cortical and brainstem regions, these associations may reflect descending modulatory activity of the mid-insula, in the context of allergen challenge, that is influenced by input from the anterior insula and prefrontal cortex [28], [29], [31], [34]. Visceromotor activity arising from the mid-insula has been shown previously. In rats, stimulation of this region causes various autonomic-driven effects, including changes in heart rate, blood pressure, respiration and gastric motility [31], [35], [36]. Although substantial evidence suggests that important differences exist in regional insula function in sub-primates compared to primates, stimulation and epileptic activity in the human insula confirms visceromotor function in the human insula, though the precise region is unclear [35].

The anterior insula is highly connected to regions of prefrontal cortex that have been shown to play an important role in emotion regulation [28], [29]. Thus, this may be a region important in the integration of cognitive and emotional activity, i.e. providing the cognitive backdrop, with changes in peripheral physiology. Indeed, activity in the anterior insula has been shown to predict the subjective perception of sensory stimulation, rather than the objective intensity of the stimulus [37], [38]. Lesions to this area tend to disrupt the unpleasantness associated with sensory experience, but not the perception of intensity [39]. In our dataset, increased activity in the anterior insula was seen selectively in response to disease-relevant cognitive stimuli prior to the development of a late-phase response. Consistent with its patterns of connectivity, magnitude of activity in this region may be a function of the intensity of interoceptive signals coming from more posterior insula regions and of activity related to cognitive and emotional factors arising from prefrontal cortical input. Extant evidence [40] suggests that given certain cognitive cues or emotional priming, homeostatic signals become amplified, or given certain homeostatic changes, cognitive cues and emotional factors may increase in salience, the integration of which is reflected in anterior insula activity. Since the magnitude of homeostatic signals, indicated by identical early phase responses to allergen, did not differ between our two asthma groups, the tendency to develop a late-phase response may depend on this process of integration, i.e. the neural context. Indeed, anterior insula activity also predicted the future increase in inflammation as reflected by EOS in sputum, which suggests that the relative gain introduced during the integration of cognitive cues with afferent input, may have been influential in the development of the late-phase response.

Though these data cannot address the origin of these group differences, they do point to fruitful directions for future research. If the late phase response to allergen depends, at least in part, on neural responsivity to disease-relevant information, then reducing this responsivity should decrease the magnitude of the late-phase response. Diminished neural responsivity to peripheral disease-related sensory signaling, via cognitive influence, has been demonstrated. Price and colleagues [41] reported that neural activity in the insula, during rectal distention in individuals with irritable bowel syndrome, was significantly reduced when placebo analgesia was administered. A similar paradigm could be employed in the context of the late-phase response. A significant reduction in airway hypersensitivity to a placebo bronchodialator has already been demonstrated in asthma [42]. If placebo were to reduce the insula response to asthma words during Ag challenge, behavioral interventions aimed at reducing neural reactivity, such as meditation [43] or neurofeedback [44] might be explored.

The small sample size in this study may limit the strength of conclusions that can be drawn. It should be noted however, that each participant completed 9 visits, which included two fMRI scans, two inhalation challenges, and two sputum inductions. Thus the complexity of the protocol and expense prohibited a larger sample. In addition, the current data conceptually replicate those observed in our previous investigation, decreasing the likelihood that they are false positives.

Overall, these data highlight the importance of specific central nervous system circuitry in the neural representation and regulation of peripheral disease-relevant processes. The fact that this circuitry is also implicated more generally in the construction of an overall sense of subjective state [45], suggests a potential mechanism underlying the increased rates of psychopathology among individuals with asthma. It has long been postulated that interoceptive information is at least influential in, if not an inextricable component of emotional experience [27], [46], [47], [48]. Therefore this experimental paradigm may be useful in identifying individuals most vulnerable to co-morbid psychopathology or those more likely to benefit from interventions targeted to reduce neural responsivity.

## Supporting Information

Figure S1 **Percent signal change in anterior insula for each valence and challenge condition.**
(PDF)

Figure S2 **Insula activity predicts peripheral measures of inflammatory potential in asthmatic participants.**
(PDF)

Figure S3 **a & b: Insula activity predicts magnitude of airway obstruction and cognitive interference of disease-related information during the late phase response to Ag in asthmatic participants.**
(PDF)

Figure S4 **Greater posterior insula activity in asthmatics (collapsed across groups) than controls, across valence, during the Ag challenge.**
(PDF)

Table S1 **Results of repeated measures ANOVA.**
(DOCX)

Table S2 **Results of voxel-wise regressions.**
(DOCX)

Supplementary Information S1 **Missing Data.**
(DOCX)

Supplementary Information S2 **Correction for multiple comparisons.**
(DOCX)

Supplementary Information S3 **Dorsal vs. ventral posterior insula.**
(DOCX)
