# Supplementary material for: Are There Neurophenotypes for Asthma? Functional Brain Imaging of the Interaction between Emotion and Inflammation in Asthma
Source: PLoS One. 2012 Aug 1;7(8):e40921. doi: 10.1371/journal.pone.0040921 (PMC3411610; doi:10.1371/journal.pone.0040921)
Supplement: Figure S1 — Percent signal change in anterior insula for each valence and challenge condition. (PDF) [file pone.0040921.s001.pdf]

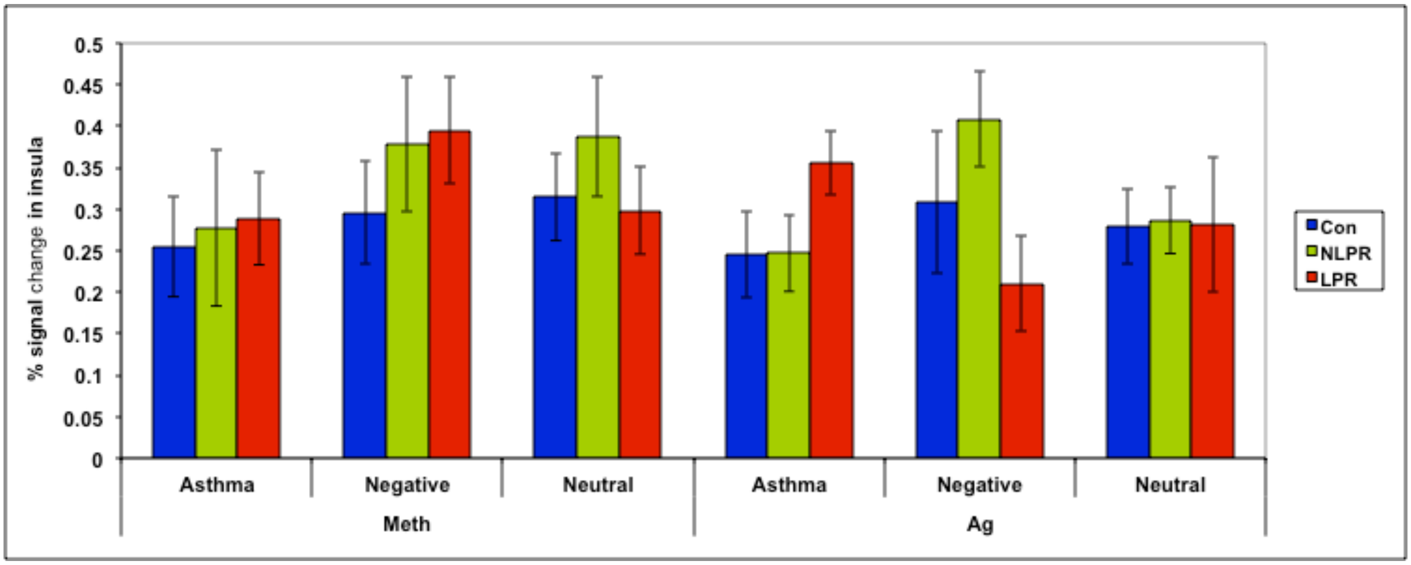

Figure 2: Percent signal change in anterior insula for each valence and challenge condition. The difference in right anterior insula response to asthma-related versus negative words, during antigen versus methacholine challenge (Ag(As-Ng)-Meth(As-Ng)) in the late phase group was significantly different from the insula response of both the non-late phase group ($F(1,15) = 6.47$, $p = .02$) and the control group ($F(1,17) = 10.53$, $p = .005$). The overall group x challenge x valence interaction was significant ($F(4, 48) = 3.24$, $p < .05$). Error bars represent standard error of the mean. Coordinates are in Montreal Neurological Institute Space (MNI).
