# Supplementary material for: Are There Neurophenotypes for Asthma? Functional Brain Imaging of the Interaction between Emotion and Inflammation in Asthma
Source: PLoS One. 2012 Aug 1;7(8):e40921. doi: 10.1371/journal.pone.0040921 (PMC3411610; doi:10.1371/journal.pone.0040921)
Supplement: Figure S2 — Insula activity predicts peripheral measures of inflammatory potential in asthmatic participants. (PDF) [file pone.0040921.s002.pdf]

a.

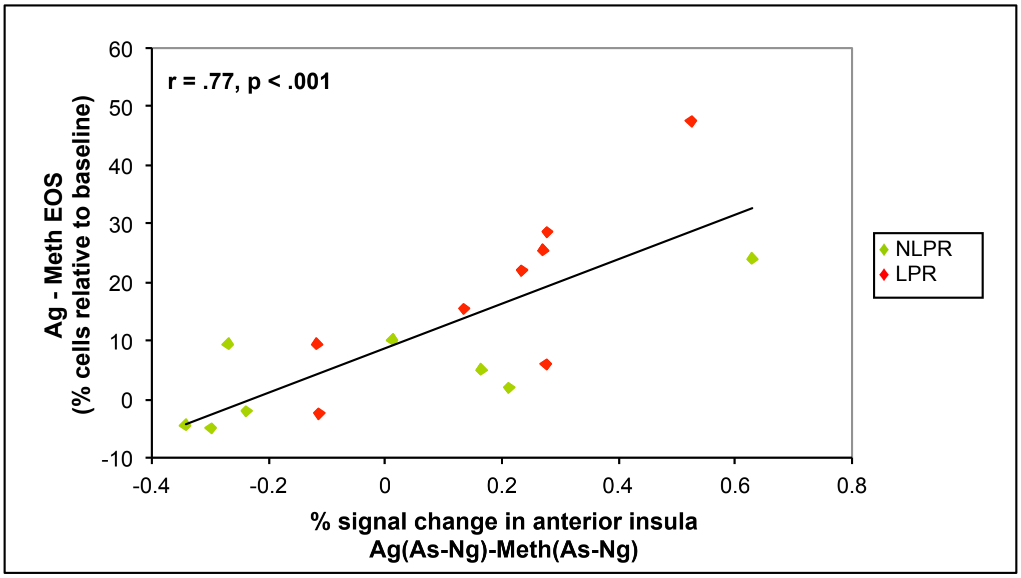

b.

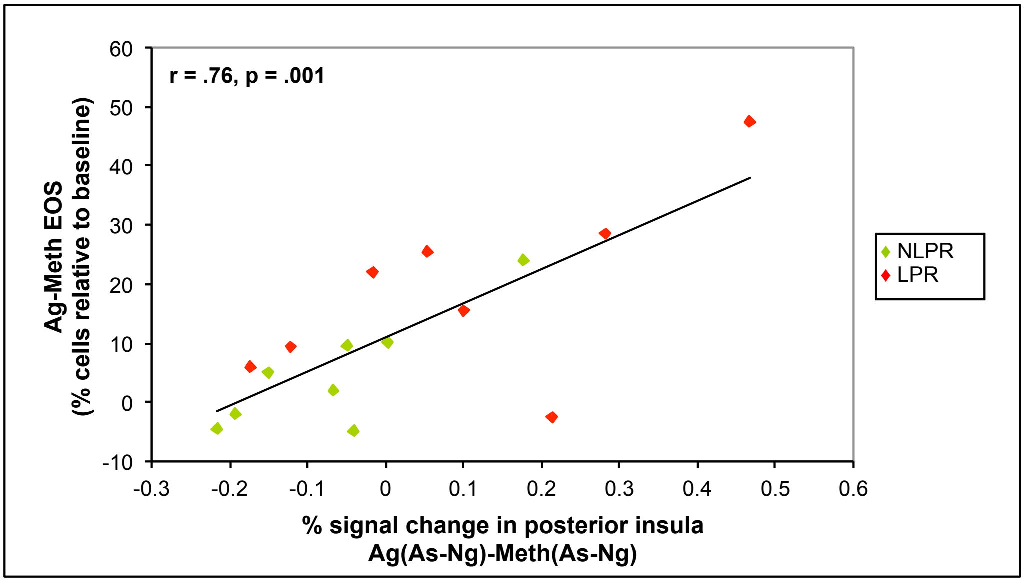

Figure 3: Insula activity predicts peripheral measures of inflammatory potential in asthmatic participants.

Percent signal change in the (a) anterior (r = .77, p < .001) and (b) posterior (r = .76, p = .001) insula in response to asthma, compared to negative words, during antigen, relative to methacholine challenge (Ag[As-Ng]-Meth[As-Ng]) and percentage of EOS in sputum during late phase antigen relative to methacholine challenge [Ag-Meth].
