# Supplementary material for: Are There Neurophenotypes for Asthma? Functional Brain Imaging of the Interaction between Emotion and Inflammation in Asthma
Source: PLoS One. 2012 Aug 1;7(8):e40921. doi: 10.1371/journal.pone.0040921 (PMC3411610; doi:10.1371/journal.pone.0040921)
Supplement: Figure S3 — a & b: Insula activity predicts magnitude of airway obstruction and cognitive interference of disease-related information during the late phase response to Ag in asthmatic participants. (PDF) [file pone.0040921.s003.pdf]

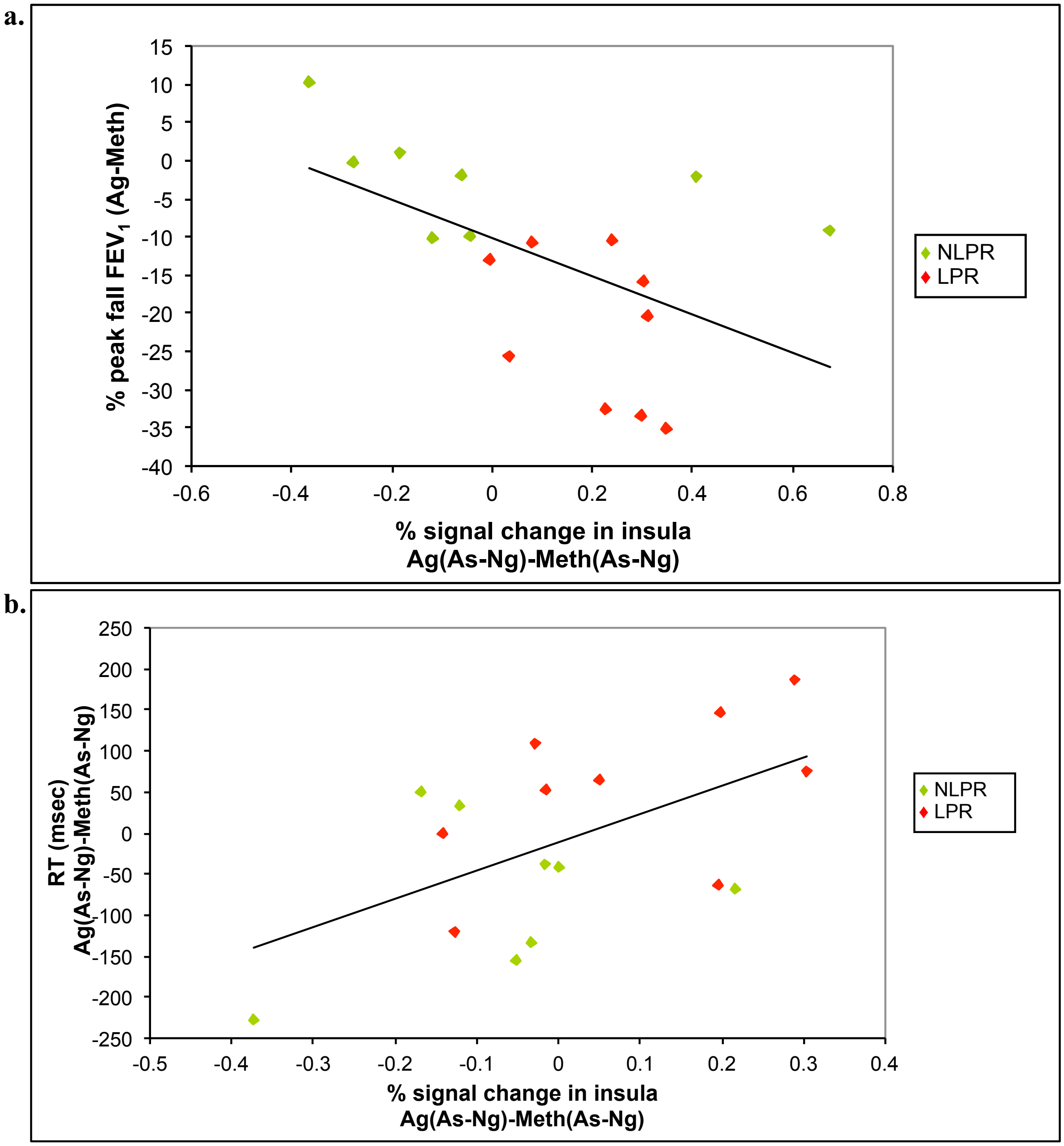

Figure 4: Insula activity predicts magnitude of airway obstruction and cognitive interference of disease-related information during the late phase response to Ag in asthmatic participants.

Percent signal change in the right mid- and posterior insula in response to asthma compared to negative words [As-Ng] and (a) percent peak fall in FEV_1 [Ag-Meth] (r = -.53, p = .03) and (b) reaction time to identify the color of As words, relative to Ng words

[Ag(As-Ng)-Meth(As-Ng)] ($r = .55$, $p < .05$) during late phase antigen relative to methacholine challenge.
