# Supplementary material for: Are There Neurophenotypes for Asthma? Functional Brain Imaging of the Interaction between Emotion and Inflammation in Asthma
Source: PLoS One. 2012 Aug 1;7(8):e40921. doi: 10.1371/journal.pone.0040921 (PMC3411610; doi:10.1371/journal.pone.0040921)
Supplement: Figure S4 — Greater posterior insula activity in asthmatics (collapsed across groups) than controls, across valence, during the Ag challenge. (PDF) [file pone.0040921.s004.pdf]

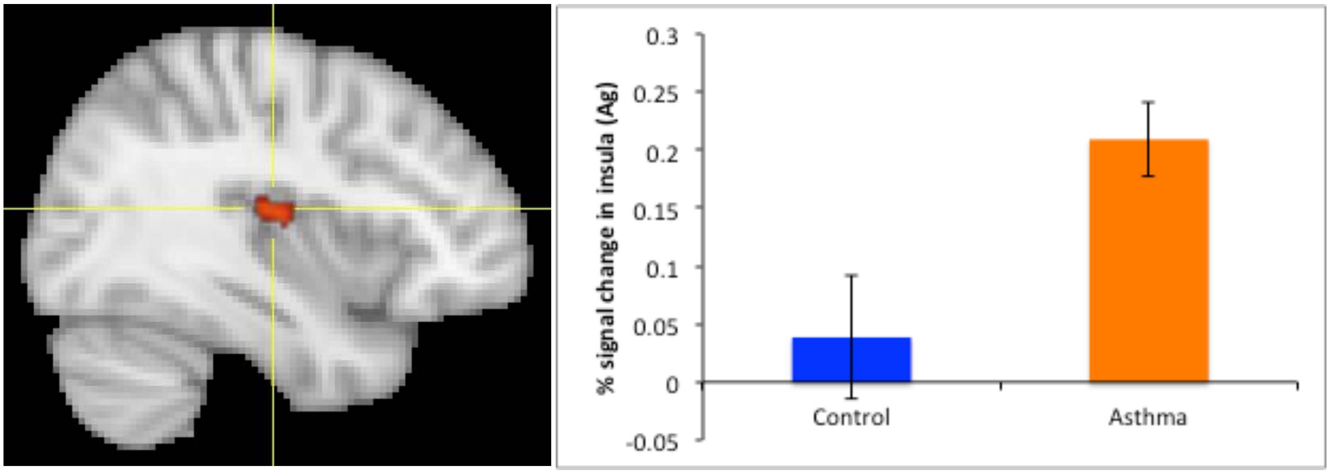

Figure 1: Posterior insula is more active in individuals with asthma (collapsed across group) than controls during Ag challenge ($t(25) = -2.95$, $p < .01$).
