# Supplementary material for: Are There Neurophenotypes for Asthma? Functional Brain Imaging of the Interaction between Emotion and Inflammation in Asthma
Source: PLoS One. 2012 Aug 1;7(8):e40921. doi: 10.1371/journal.pone.0040921 (PMC3411610; doi:10.1371/journal.pone.0040921)
Supplement: Table S1 — Results of repeated measures ANOVA. (DOCX) [file pone.0040921.s005.docx]

**Table S1:** Results of repeated measures ANOVA

| **Region** | **Contrast** | **Cluster volume (mm^3^)** | **Cluster p-value (corrected)** | **Coordinates of cluster peak** | **Peak voxel F-value** |
| --- | --- | --- | --- | --- | --- |
| anterior insula | Group x challenge x valence | 616 | .119 | 44, 13, -8 | 4.51 |
|  | Group x challenge | 312 | .622 | -45, 15, -1 | 7.93 |
| ventral posterior insula | Group x challenge x valence | 1496 | .002 | -45, -30, 4 | 4.65* |
| inferior frontal gyrus | Group x challenge x valence | 904 | .003 | -47, 27, 5 | 5.83 |
| middle frontal gyrus | Group x challenge | 2080 | < .001 | 32, 51, 1 | 10.00 |
| thalamus | Group x challenge | 520 | < .001 | 6, -34, 5 | 23.69 |
|  | Group x challenge | 424 | < .001 | -6, -34, 7 | 15.63 |
| caudate | Group x challenge | 528 | < .001 | 6, 1, 5 | 15.6 |

These clusters were identified using a 3 (group) x 2 (challenge) x 3 (valence) mixed model repeated measures ANOVA with subject as a random factor. Monte Carlo simulation was used to correct for multiple comparisons, based on volumetric criteria. First, clusters were identified using a voxel-wise threshold of two-tailed p ≤ 0.05 uncorrected in the a priori insula and ACC regions of interest, and p ≤ 0.01 uncorrected in all other regions. Next, corrected cluster p-values were identified by computing the probability of obtaining equivalently sized clusters from random noise, based on 1000 simulations. For regions outside the focus of our a priori predictions, an average whole brain of all participants comprised the search volume for simulations. P-values for insular and ACC clusters were corrected for multiple comparisons within insular and ACC masks respectively. Coordinates are reported in Montreal Neurological Institute (MNI) Space. *Note the voxel with peak activation in this cluster is actually located on the edge of the superior temporal gyrus, though the vast majority of voxels in this cluster are located in the insula.
