# Supplementary material for: Are There Neurophenotypes for Asthma? Functional Brain Imaging of the Interaction between Emotion and Inflammation in Asthma
Source: PLoS One. 2012 Aug 1;7(8):e40921. doi: 10.1371/journal.pone.0040921 (PMC3411610; doi:10.1371/journal.pone.0040921)
Supplement: Table S2 — Results of voxel-wise regressions. (DOCX) [file pone.0040921.s006.docx]

**Table S2:** Results of voxel-wise regressions

| **Region** | **Voxel-wise correlation with** | **Cluster volume (mm^3^)** | **Cluster p-value (corrected)** | **Coordinates of cluster peak** | **Peak voxel t-value** |
| --- | --- | --- | --- | --- | --- |
| anterior insula/superior temporal gyrus | Ag-Meth EOS | 1896 | < .001 | 44, 7, -12 | 4.01 |
| posterior insula | Ag-Meth EOS | 432 | .001 | 51 -28 16 | 3.37 |
| mid-insula | Ag-Meth FEV | 560 | < .001 | 51, -7, 8 | -3.43* |
| mid- to posterior insula | Ag(As-Ng)-Meth(As-Ng) RT | 4472 | < .001 | 40, -2, 19 | 4.33 |
| medial frontal gyrus | Ag-Meth EOS | 1336 | < .001 | -14, -19, 58 | 5.52 |
| postcentral gyrus | Ag-Meth EOS | 2192 | < .001 | 67, -17, 14 | 4.90 |
| precuneus | Ag-Meth EOS | 5368 | < .001 | 2, -70, 38 | 4.97 |
| superior frontal gyrus | Ag(As-Ng)-Meth(As-Ng) RT | 1536 | < .001 | 14, 43, 57 | -4.58 |

These clusters were identified using whole-brain voxel-wise regressions, where percent signal change in response to asthma words, relative to negative words, during antigen, compared to methacholine challenge (Ag(As-Ng) – Meth(As-Ng)) was used to predict the metric of peripheral biology or behavior indicated. A voxel-wise threshold of p ≤ 0.01 uncorrected was used, when identifying clusters throughout the brain. After clusters were identified at the given thresholds, Monte Carlo simulation was used to correct for multiple comparisons. Coordinates are reported in Montreal Neurological Institute (MNI) Space.

* Note that the peak voxel in this cluster is most likely located in the precentral gyrus, though the majority of the cluster is located in the insula, with a second peak at (40, -3, 15).
