# Supplementary material for: Are There Neurophenotypes for Asthma? Functional Brain Imaging of the Interaction between Emotion and Inflammation in Asthma
Source: PLoS One. 2012 Aug 1;7(8):e40921. doi: 10.1371/journal.pone.0040921 (PMC3411610; doi:10.1371/journal.pone.0040921)
Supplement: Supplementary Information S1 — Missing Data. (DOCX) [file pone.0040921.s007.docx]

**Missing data**

Lung function data were not available for one participant in the control group. Sputum data was unavailable for one participant in the LPR group. Reaction time data were excluded from analysis for two participants (1 LPR, 1 control) because greater than 25% of their responses were incorrect. fMRI data from one individual in the LPR group were excluded due to excessive motion.
