# Supplementary material for: Are There Neurophenotypes for Asthma? Functional Brain Imaging of the Interaction between Emotion and Inflammation in Asthma
Source: PLoS One. 2012 Aug 1;7(8):e40921. doi: 10.1371/journal.pone.0040921 (PMC3411610; doi:10.1371/journal.pone.0040921)
Supplement: Supplementary Information S2 — Correction for multiple comparisons. (DOCX) [file pone.0040921.s008.docx]

**Correction for Multiple Comparisons**

First, clusters were identified using a voxel-wise threshold of two-tailed p ≤ 0.05 (uncorrected) in the a priori (insula and ACC) regions of interest, and p ≤ 0.01 (uncorrected) in all other regions. Next, corrected cluster p-values were identified by computing the probability of obtaining equivalently sized clusters from random noise, based on 1000 simulations. For regions outside the focus of our a priori predictions, an average whole brain of all participants comprised the search volume for simulations. P-values for insular and ACC clusters were corrected for multiple comparisons within insular and ACC masks, respectively.
