# Supplementary material for: Are There Neurophenotypes for Asthma? Functional Brain Imaging of the Interaction between Emotion and Inflammation in Asthma
Source: PLoS One. 2012 Aug 1;7(8):e40921. doi: 10.1371/journal.pone.0040921 (PMC3411610; doi:10.1371/journal.pone.0040921)
Supplement: Supplementary Information S3 — Dorsal vs. ventral posterior insula. (DOCX) [file pone.0040921.s009.docx]

**Dorsal vs. ventral posterior insula**

We distinguish activity in the dorsal aspect of the posterior insula from activity in the ventral aspect, based on recent observations (1). These observations delineate distinct differences in cytoarchitecture in human post-mortem brain between these two regions, which suggest differences in function. In the current investigation, we observed greater activation of the ventral posterior insula in response to Ng words relative to As or Ne, during Ag challenge compared in Meth, in the NLPR group. This cluster of activation coincides perfectly with the dysgranular region of the posterior insula, described by Kurth and colleagues (1). Previous anatomical studies (2, 3) did not distinguish dorsal and ventral insular regions and as a consequence, no distinction has been made with regard to reporting functional significance of activity in this region. Clusters of activation appearing in this ventral posterior region have been observed during the experiences of hypercapnia (4) and anxiety (5), but its specific contribution to these experiences is yet unknown.

**References**

1. Kurth F, Zilles K, Fox PT, Laird R & Eickhoff SB (2010) A link between the systems: functional differentiation and integration within the human insula revealed by meta-analysis. Brain Struct Funct 214: 519–534.

2. Mesulam, E.J. & Mufson, M.-M (1982a). Insula of the old world monkey. III: Efferent cortical output and comments on function. J. Comp. Neurol. **212**, 38-52.

3. Friedman, D.P., Murray, E.A., O’Neill, J.B. & Mishkin, M. (1986). Cortical connections of the somatosensory fields of the lateral sulcus of macaques: Evidence for a corticolimbic pathway for touch. *J. Comp. Neurol.* **252**, 323-327.

4. Harper, R.M. et al. (2005). Hypercapnic exposure in congenital central hypoventilation syndrome reveals CNS respiratory control mechanisms. *J. Neurophysiol.* **93**, 1647-1658.

5. Liotti, M. et al. (2000). Differential limbic--cortical correlates of sadness and anxiety in healthy subjects: implications for affective disorders. *Biol. Psychiatr.* **48**, 30-42.
